# Society Meetings

**Published:** 1897-01

**Authors:** 


### Society Meetings.

The Medical Society of the State of New York will hold its ninety-first annual meeting at Albany, Tuesday, Wednesday and Thursday, January 26th, 27th and 28th, 1897, under the presidency of Dr. James D. Spencer, of Watertown. The other officers are: Vice-president, Dr. L. Duncan Bulkley; secretary, Dr. Frederick C. Curtis, 17 Washington avenue, Albany, N. Y.; treasurer, Dr. Charles H. Porter, 103 Lancaster street, Albany. The business committee is composed as follows: Seneca D. Powell, chairman, 12 West 40th street, New York; Willis G Macdonald, 27 Eagle street, Albany; Ernest Wende, 471 Delaware avenue, Buffalo. Chairmen of standing committees: arrangements, William J. Nellis, Albany; on by-laws, H. D. Wey, Elmira; on hygiene, Henry R. Hopkins, Buffalo; on legislation, A Walter Suiter, Herkimer; on ethics, Charles Jewett, Brooklyn; on prize essays, Abraham Jacobi, New York; of publication, F. C. Curtis, Albany.

Up to the time of going to press with this edition of the Journal, December 24th, the provisional program had not been issued, but certain features of it have been made known from which we group the following:

There will be three general discussions of importance: one pertaining to medicine, one to surgery, and another to obstetrics. The surgical discussion exclusively devoted to the field of rectal surgery, will be led by Professor Joseph M. Mathews, M. D., of

Louisville, Ky., the well-known author and teacher in this department. Other participants will be Drs. Daniel Lewis, Charles B. Kelsy and Robert T. Morris, of New York ; George R. Fowler, of Brooklyn, and Edward Clark, of Buffalo.

The medical discussion will be devoted to the relation of impure drinking water to disease, and will include the cure and prevention of disease. These will be considered under the following heads :

(1) Common causes of the contamination of drinking water : (*a*) mineral ; (*b*) living organisms : from soil, from intestinal tracts of man, from urinary tracts of man ? (2) Diseases which can be directly traced to contaminated drinking water : (*a*) mineral poisons ; (*b*) bacterial and protozoon infection: soil, malaria, feces, urine, diarrhea, cholera, typhoid, fever, etc. ? (3) Dangers of the domestic use, other than drinking, of contaminated water. (4) Methods of purification of impure water. (5) Methods of prevention of pollution of water. (6) Bacteriological diagnosis of typhoid fever. (7) Typhoid fever in children. (8) Treatment of typhoid fever : (*a*) medicinal and dietetic ; (*b*) external and internal use of water ; importance and practical methods of disinfecting the excreta from typhoid patients.

Dr. Charles G. Stockton, of Buffalo, will present the subject (*b*) section 8, The value of external and internal use of water in typhoid fever. Other participants will be Drs. T. M. Cheesman, J. S. Billings, G. R. Freeman, A. H. Ely, W. G. Thompson, of New York ; G. A. Blumer, Utica ; Thomas B. Carpenter, Buffalo ; F. C. Curtis, Albany ; W. S. Ely, Rochester.

The obstetric discussion will be as follows : (1) Introduction : Puerperal eclampsia, by William Warren Potter, of Buffalo. (2) Indication for, and technique of, the induction of premature labor, by James P. Boyd, Albany. (3) Infection of the puerpera, by A. B. Miller, Syracuse. (4) Obstetric delivery by abdominal section : indications and methods, by W. G. Macdonald, Albany. (5) Subject to be announced, by William M. Polk, New York. (6) Relations of posterior displacements to pregnancy : treatment by a new method, by M. D. Mann, Buffalo. (7) Present treatment of fibroids associated with pregnancy, by A. Vander Veer, Albany.

The usual railroad rates will be secured, and it is expected that the attendance will be large. Hotel accommodations should be secured by letter in advance, as Albany is generally crowded with people in midwinter.

It is important that the delegates from the Medical Society of the County of Erie should attend these meetings in full force and with regularity.

---

THE Buffalo Academy of Medicine held meetings during December, 1896, as follows :

*Section on Surgery.*—Tuesday evening, December 1st, with the following program : A brief review of the general technique of certain cranio-cerebral operations, by Dr. C. B. Nancrede, of Ann Arbor, Professor of Surgery, University of Michigan. Presentation of cases : I. Nerve anastomosis ; II. Removal of Meckel's ganglion for neuralgia ; III. Wound of occipital sinus—trephined, by Dr. Roswell Park ; IV. A case, by Dr. Eugene A. Smith ; V. Gunshot wound of forehead—localisation of bullet by Girdner's telephone bullet probe, trephined occipital region, bullet removed, demonstration of bullet probe, by Dr. William C. Phelps ; VI. Three cases by Dr. E. J. Myers.

*Section on Medicine.*—Tuesday evening, December 8th. Program : Care of delicate children, by Dr. —— —— Ross ; Feeding of children under five years of age, by Dr. DeLancey Rochester ; Serum diagnosis of typhoid fever, report on a series of fifty cases, by Dr. Frank H. Thornbury and Dr. A. H. Appell. A confirmatory report of Widal's serum diagnosis of typhoid fever, Dr. Julius Ullman and Dr. Albert E. Woehnert. Report of an unusual clinical case, Dr. G. A. Himmelsbach.

*Section on Pathology.*—Tuesday evening, December 15th, program : Exhibition of gynecological specimens, by Dr. H. E. Hayd ; Perforation of the uterus, by Dr. Sidney A. Dunham ; Perforating ulcer of the intestine, by Dr. N. G. Russell ; Comparative anatomy of the cecum and appendix vermiformis in a dog, by A. T. Kerr, Jr.

*Section on Obstetrics and Gynecology.*—Tuesday evening, December 22d. Program : Uterine displacements : etiology and pathology, by Dr. M. A. Crockett ; Diagnosis and treatment, by Dr. Stephen Y. Howell.

---

THE TWELFTH INTERNATIONAL MEDICAL CONGRESS will be held at Moscow, Russia, August 19-26, 1897. The American national committee consists, according to the directions of the general committee, at Moscow, of the following-named gentlemen: J. S. Billings, M. D., New York; F. J. Shepherd, M. D., Montreal; Geo. B. Shattuck, M. D., Boston; W. S. Thayer, M. D., Baltimore; Geo. F. Shrady, M. D., New York; Frank P. Foster, M. D., New York; Charles A. L. Reed, M. D., Cincinnati; S. Weir Mitchell, M. D., Philadelphia; A. Jacobi, M. D., chairman, 110 West 34th street, New York.

The chairman begs to invite the attention of the medical profession of the United States and Canada to the fact that the professional gentlemen in charge of the congress are anxious to make it a success, both from a scientific and social point of view.

Their difficulties are unusually grave; but it is not their fault that the congress had to wait for governmental permission to meet in their country, or that a special ukase was required for the admission into Russia of Jewish medical men on equal terms with their Greek, Catholic, Protestant, Agnostic and Mohammedan colleagues, or that the famous and meritorious Secretary-General was—it appears because of his liberalism—ousted both from his place and from his professorship. They should not be held responsible for the political semi-barbarism of the country in which they live and to whose laws they have to submit. Their position in the world of science, and their endeavor to make the twelfth congress equal to its most famous predecessors, will prove an incentive to American physicians to sustain, both by their presence and their contributions, the Russian committee in its exertions to make the next congress equal to its predecessors.

Such information as may be received from time to time will be published in the medical journals immediately after its arrival.

---

THE Medical Society of the County of Erie will hold its seventy-sixth annual meeting Tuesday, January 12, 1897, at the rooms of the Buffalo Academy of Medicine, in the Market Arcade, under the presidency of Dr. J. G. Thompson, of Angola, N. Y. The president is desirous of giving considerable attention to the program which has been prepared for the occasion, and for that reason will open the proceedings promptly at 10 o'clock A. M. The papers to be presented are all of interest to the general practitioner of medicine, and free discussion is invited.

Dr. P. W. Van Peyma will read a paper on Albuminuria of pregnancy; Dr. F. W. Hinkel, will follow with one entitled, Earache, its significance and management; and finally Dr. Thomas

B. Carpenter will present the subject of Genito-urinary epithelium. A full attendance of members is respectfully urged by the officers.

---

THE Medical Society of the County of Chautauqua will hold its semi-annual meeting at the Hotel Columbia, Fredonia, N. Y., January 12, 1897, under the presidency of Dr. E. S. Rich, of Kennedy. The secretary, Dr. C. A. Ellis, has sent out the following program :

*Appendicitis*—1. Anatomy, paper, by Dr. N. E. Beardsley, Dunkirk. 2. Etiology, paper, by Dr. N. G. Richmond, Fredonia. 3. Symptoms and differential diagnosis, paper, by Dr. V. M. Griswold, Fredonia ; discussed by Drs. Wm. C. Duke, Cassadaga, and W. Stuart, Westfield. 4. Medicinal treatment, paper, by Dr. John C. Lewis, Panama ; discussed by Drs. G. E. Ellis, Brocton, and O. C. Shaw, Kennedy. 5. Surgical treatment, paper, by Dr. John Parmenter, University of Buffalo, Buffalo, N. Y. ; discussed by Drs. E. M. Scofield, Jamestown, and R. T. Rolph, Dunkirk. General discussion and report of cases.

A cordial invitation is extended to the profession of adjoining counties to attend and take part in all discussions.

---

THE Western Ophthalmological, Otological, Laryngological and Rhinological Association will meet at St. Louis, Mo., the second Thursday and Friday of April, 1897. Physicians desiring to read papers are invited to send subjects to the secretary, Dr. Hal Foster, Kansas City, at once. Railways will give one and one-third fares on the certificate plan. Programs will be issued February 1, 1897. The profession is cordially invited to attend.

